# An Optimized SYBR Green I/PI Assay for Rapid Viability Assessment and Antibiotic Susceptibility Testing for *Borrelia burgdorferi*

**DOI:** 10.1371/journal.pone.0111809

**Published:** 2014-11-03

**Authors:** Jie Feng, Ting Wang, Shuo Zhang, Wanliang Shi, Ying Zhang

**Affiliations:** Department of Molecular Microbiology and Immunology, Bloomberg School of Public Health, Johns Hopkins University, Baltimore, Maryland, United States of America; University of Toledo School of Medicine, United States of America

## Abstract

Lyme disease caused by *Borrelia burgdorferi* is the most common tick-borne disease in the US and Europe. Unlike most bacteria, measurements of growth and viability of *B. burgdorferi* are challenging. The current *B. burgdorferi* viability assays based on microscopic counting and PCR are cumbersome and tedious and cannot be used in a high throughput format. Here, we evaluated several commonly used viability assays including MTT and XTT assays, fluorescein diacetate assay, Sytox Green/Hoechst 33342 assay, the commercially available LIVE/DEAD BacLight assay, and SYBR Green I/PI assay by microscopic counting and by automated 96-well plate reader for rapid viability assessment of *B. burgdorferi*. We found that the optimized SYBR Green I/PI assay based on green to red fluorescence ratio is superior to all the other assays for measuring the viability of *B. burgdorferi* in terms of sensitivity, accuracy, reliability, and speed in automated 96-well plate format and in comparison with microscopic counting. The BSK-H medium which produced a high background for the LIVE/DEAD BacLight assay did not affect the SYBR Green I/PI assay, and the viability of *B. burgdorferi* culture could be directly measured using a microtiter plate reader. The SYBR Green I/PI assay was found to reliably assess the viability of planktonic as well as biofilm *B. burgdorferi* and could be used as a rapid antibiotic susceptibility test. Thus, the SYBR Green I/PI assay provides a more sensitive, rapid and convenient method for evaluating viability and antibiotic susceptibility of *B. burgdorferi* and can be used for high-throughput drug screens.

## Introduction

Lyme disease is a tick-borne illness caused by the spirochete *Borrelia burgdorferi* [1], [2]. The disease is transmitted by tick vectors that could spread by rodents, reptiles, birds and deer [1], [2]. In the United States, Lyme disease is considered the most common tick-borne disease and the number of Lyme disease cases has been increasing in the last 15 years [1], [2]. Clinical and experimental studies have observed various morphologic forms of *B. burgdorferi*: spirochete, spheroplast (or L-form), cystic or round body forms [3]–[5], and biofilm-like colonies [6]. The currently used frontline drugs such as doxycycline and amoxicillin while showing good activity for growing spirochetal form of *B. burgdorferi* have limited activity on variant forms or biofilm or persister forms of *B. burgdorferi* [6], [7], which may underlie persistent symptoms that occur in some patients after standard treatment.

Unlike most bacteria such as *E. coli* and *S. aureus* that form clearly visible colonies on agar plates, most *B. burgdorferi* grows poorly as colonies on agar plates, which makes precise quantification of its growth difficult. In liquid culture, *B. burgdorferi* cells do not provide adequate light absorption for direct optical density measurement. Lack of a convenient viability testing method for *B. burgdorferi* has hampered antibiotic susceptibility testing and new drug discovery for this organism. Currently, microscopic counting and quantitative PCR (qPCR) or reverse-transcription PCR (RT-PCR) are the main assays for rapid assessment of *B. burgdorferi* viability. Manual microscopic counting using counting chamber is mainly used to assay *B. burgdorferi* viability *in vitro*, but it is very time-consuming and is hard to perform in microtiter plate. Although quantitative PCR (qPCR) could detect very little DNA and is usually applied for *B. burgdorferi* viability assay *in vivo,* it is complex, costly, and not suitable for viability assay of bacteria in high-throughput drug screen format. Several rapid colorimetric assays have been used to determine bacterial viability in general: fluorescein diacetate (FDA) assay [8], [9], XTT (2,3-bis-(2-methoxy-4-nitro-5-sulfophenyl)-2h-tetrazolium-5-carboxanilide) assay [10], MTT (3-(4,5-dimethylthiazol-2-yl)-2,5-diphenyltetrazolium bromide) assay [11], [12], fluorogenic dye SYTO 9 (LIVE/DEAD) assay [13], [14], Sytox Green/Hoechst 33342 assay [15], and SYBR Green assay [16], [17]. The LIVE/DEAD BacLight assay has been used as a gold standard to measure the viability and antibiotic susceptibility of *B. burgdorferi* [3], [6], [18]. However, these assays either have limited sensitivity or are cumbersome and cannot be performed in a high throughput format. In the case of SYBR Green I assay, it has not been tested on *B. burgdorferi* as a viability assay.

In an attempt to find a more rapid and sensitive viability assay that can be used for antibiotic susceptibility tests and for high throughput drug screens to identify new drugs for *B. burgdorferi* persisters for improved treatment of Lyme disease, here we compared the above methods for quantification of *B. burgdorferi* viability in a 96 well microtiter plate format amenable to high throughput screens of drugs and also optimized the SYBR Green I/propidium iodide (PI) assay. We found the optimized SYBR Green I/PI assay to be superior to the other assays including the commercial LIVE/DEAD BacLight assay for rapidly assessing *B. burgdorferi* viability in a high throughput format. This new SYBR Green/PI assay will be useful for antibiotic susceptibility testing of *B. burgdorferi* and has recently been shown to be a powerful method for identifying drugs against *B. burgdoferi* persisters from an FDA-approved drug library in a high througput format [7].

## Materials and Methods

### Strain, media and culture

*B. burgdorferi* B31 strain was obtained from American Type Tissue Collection (ATCC 35210). *B. burgdorferi* was cultured in BSK-H medium (HiMedia Laboratories Pvt. Ltd.) containing 6% rabbit serum (Sigma-Aldrich). All culture media were filter-sterilized by 0.2 µm filter. Cultures were incubated in sterile 50 ml closed conical tubes (BD Biosciences, California, USA) at 33°C without antibiotics. After 5 days, *B. burgdorferi* culture (1×10^6^ cells) was transferred into 96-well tissue culture microplate for testing by different methods (see below) for viability measurement.

The method for preparation of *B. burgdorferi* biofilm using 96-well tissue culture plates was performed as described [4]. Briefly, for generation of *B. burgdorferi* biofilm, spirochetes *B. burgdorferi* were cultured in 96-well plates (BD BioCoat Multiwell Plates) for 10 days without shaking. After the 10 days of incubation, biofilm-like colonies were examined by inverted microscope. After the biofilm formation the supernatant was removed and the wells were rinsed with 100 µl fresh BSK-H medium. Subsequently 100 µl fresh BSK-H medium was added to each well for viability measurement (see below).

### Microscopy techniques

Specimens of *Borrelia* cultures or cells were examined on a Nikon Eclipse E800 microscope equipped with differential interference contrast (DIC) and epifluorescence illumination, and recorded with a Spot slider color camera. Biofilm of *B. burgdorferi* in the 96-well plate was visualized using the Nikon TE200 inverted microscope equipped with epifluorescence, and recorded by a monochrome camera. Zeiss AxioImager M2 microscope equipped with ORCA-R^2^ high resolution digital camera (HAMAMATSU, Japan) was used to observe spirochetes stained by Sytox Green/Hoechst 33342 assay (see below).

### MTT, XTT, and FDA assays

*B. burgdorferi* cultures were placed in 96-well microtiter plate followed by addition of 20 µl of 5 mg/ml MTT in PBS or 50 µl XTT solution (1 mg/ml) in phenazine methosulfate (PMS) to each well. The plate was incubated in the dark at 33°C for 2 to 5 hours. The supernatant was removed from the wells and the formazan was dissolved in 100 µl of DMSO for 15 min or in 100 µl 10% SDS in 0.01 M HCl for 2 hours. The absorbance was measured at 565 nm for MTT assay and at 450 nm for XTT assay using HTS 7000 Plus Bio Assay Reader (Perkin Elmer Inc., USA).

For the Fluorescein diacetate (FDA) assay, FDA was dissolved in acetone at a concentration of 10 mg/ml and was used to assess the viability of *B. burgdorferi* using a modified procedure as described previously [9]. Before each assay, the stock solution was diluted into a 1∶10 FDA working solution in MOPS buffer and 10 µl FDA working solution was added to each well of the 96-well plate. Plates were incubated in the dark at 33°C for 45 min. Fluorescence at 518 nm was measured with excitation at 494 nm using HTS 7000 Plus Bio Assay Reader (Perkin Elmer Inc.).

### LIVE/DEAD BacLight assay

LIVE/DEAD BacLight assay kit was purchased from Invitrogen and the assay was performed per manufacturer’s instructions. Briefly, a working solution was prepared by adding 30 µL SYTO 9 stock solution (5 mM) and 30 µl propidium iodide (20 mM) into 1 ml filter-sterilized dH_2_O. The working solution (10 µl) was pipetted into each well and mixed thoroughly by pipetting up and down. The plate was incubated at room temperature in the dark for 15 minutes. With the excitation wavelength at 485 nm, the fluorescence intensities at 535 nm (green emission) and 635 nm (red emission) were measured using HTS 7000 Plus Bio Assay Reader (Perkin Elmer Inc.) and the ratio of green fluorescence to red fluorescence was calculated.

### Sytox Green/Hoechst 33342 assay

The Sytox Green/Hoechst 33342 assay was performed as described by Cornell et al. [15]. Briefly, 2 µl of a 1∶1 mixture of 5 mM Sytox Green and 10 mM Hoechst 33342 was added to 200 µl of *B. burgdorferi* culture in each well of a 96-well plate. After mixing, the plate was incubated at 37°C for 1.5 h and the Sytox Green (excitation 485 nm/emission 535 nm) and Hoechst 33342 (excitation 360 nm/emission 465 nm) fluorescence was measured using HTS 7000 Plus Bio Assay Reader. The blue (465 nm)/green (535 nm) fluorescence ratios were measured for each proportion of live/dead *B. burgdorferi*.

### SYBR Green I/PI assay

For assaying the live and dead cells in 96-well plates, SYBR Green I and PI were used for double staining of nucleic acids. SYBR Green I (10,000 × stock, Invitrogen) (10 µl) was mixed with 30 µl propidium iodide (20 mM, Sigma) into 1.0 ml of sterile dH_2_O and vortexed thoroughly. The staining mixture (10 µl) was added to each well and mixed thoroughly. The plate was incubated at room temperature in the dark for 15 minutes. With excitation wavelength at 485 nm, the fluorescence intensities at 535 nm (green emission) and 635 nm (red emission) were measured for each well of the plate using HTS 7000 Plus Bio Assay Reader (PerkinElmer Inc., USA). Meanwhile the *B. burgdorferi* suspensions (live and 70% isopropyl alcohol killed) in five different proportions of live:dead cells (0∶10, 2∶8, 5∶5, 8∶2, 10∶0) was mixed in wells of 96-well plate. The SYBR Green I/PI was added to each well and the green/red fluorescence ratios were measured for each proportion of live/dead *B. burgdorferi* using HTS 7000 Plus Bio Assay Reader. With least-square fitting analysis, the regression equation and regression curve of the relationship between percentage of live bacteria and green/red fluorescence ratios were obtained. The regression equation was used to calculate the percentage of live cells in each well.

The MIC (minimum inhibitory concentration) test with the SYBR Green I/PI assay was performed in 96-well microtiter plate with 10^5^ bacteria of *B. burgdorferi* in fresh BSK-H medium containing doubling concentrations (0.2–50 µg/ml) of various antibiotics doxycycline, amoxicillin, metronidazole, and vancomycin, followed by incubation at 34°C for 6 days when the degree of growth inhibition was measured by the SYBR Green I/PI assay in HTS 7000 Plus Bio assay reader as described above.

## Results and Discussion

The major impetus for this study is because the current viability assay for *Borrelia burgdorferi* including LIVE/DEAD BacLight test cannot be used for high throughput drug screens to identify drugs that target non-growing Borrelia persisters. Thus there is a need to develop such a rapid viability that is capable of this purpose. To identify an optimal method for assaying the viability of *B. burgdorferi*, we evaluated several commonly used viability assays for bacteria including MTT assay, XTT assay, fluorescein diacetate (FDA) assay, and the commercial SYTO 9 (LIVE/DEAD BacLight) assay, and the SYBR Green I/PI assay using live and dead cells mixed in known proportions (see Method for details). The results showed that except the SYBR Green I/PI assay, each of the other method had its own defect and could not quantitatively assay the viability of *B. burgdorferi* culture (Table 1).

**Table 1:** Comparison of data^1^ of different methods for assaying *B. burgdorferi* viability.

Percent of live cells	MTT^2^(OD_565_)	XTT(OD_450_)	FDA^3^(λem)	LIVE/DEAD(Ratio _Green/Red_)	Sytox/Hoechst(Ratio _Green/Blue_)	SYBR Green I/PI(Ratio _Green/Red_)
0%	0.131	2.894	26069	10.81	0.947	3.49
20%	0.132	2.894	25938	10.13	0.943	4.15
50%	0.147	3.024	26134	11.35	0.931	5.48
80%	0.161	3.051	29136	10.75	0.927	7.17
100%	0.157	2.978	30158	11.63	0.924	8.03
BSK-H	0.152	2.892	26984	11.41	0.958	2.03

1 Each value is the mean of three replicates.

2 The formazan was dissolved in 100 µL of DMSO for 15 min.

3 Plates were incubated in dark at 33°C for 45 min before being read. λem refers to fluorescence emission at 518 nm with 494 nm excitation.

### MTT, XTT, and FDA assays

**Figure 1:**
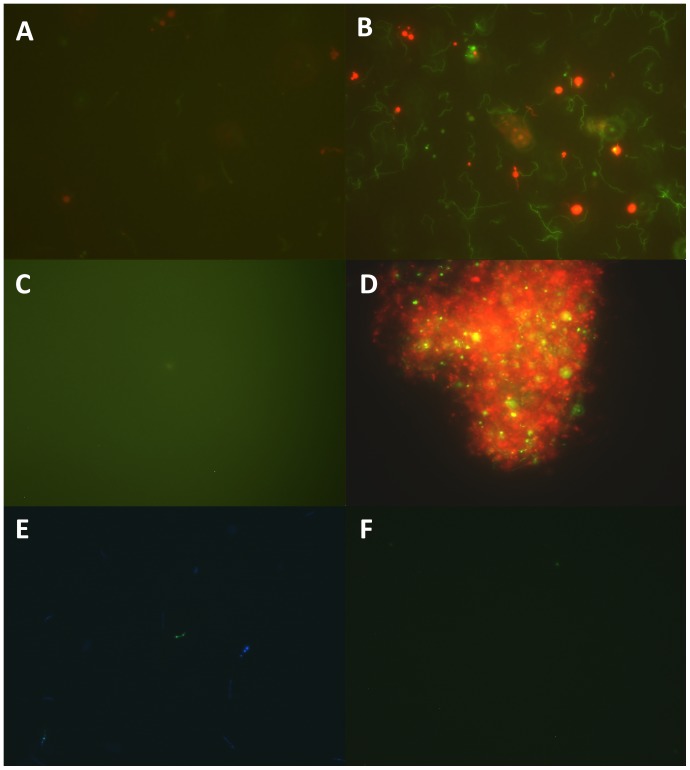
Representative images of *B. burgdorferi* culture (7 day old), observed with fluorescence microscopy equipped with Spot slider color camera using LIVE/DEAD BacLight stain (A), SYBR Green I/PI stain (B), and FDA stain (C). Antibiotic-treated *B. burgdorferi* biofilm (9 day old) was stained by SYBR Green I/PI (D). Sytox Green/Hoechst 33342 stained *B. burgdorferi* images were recorded by the ORCA-R^2^ high resolution camera (E, merged from images visualized by DAPI, FITC and TRITC filters) and by Spot slider color camera with triple filter (F).

For the MTT assay, after 5 hour incubation, the amount of purple formazan produced was still too little to produce a significant reading for colorimetric detection (Table 1). For the XTT assay, the XTT seemed to be reduced by BSK-H medium itself and produced a very strong false positive background, and the absorbance at 450 nm had no linear relationship with the percentage of live bacteria (Table 1). For the FDA assay, although the relationship between the fluorescence signals and the number of live cells could be obtained, this assay showed low reproducibility (Table 2). In addition, the linear range of the FDA assay was only between 6×10^7^ and 10^8^ cells/ml and incubation with FDA dye in the BSK medium produced strong background signal (Figure 1C). Thus the FDA assay was judged unsuitable and not sensitive enough for assessment of viability for *B. burgdorferi*.

**Table 2:** Comparison of performance of different methods for assaying *B. burgdorferi* viability.

	MTT	XTT	FDA	LIVE/DEAD	LIVE/DEAD^5^	Sytox/Hoechst	SYBR Green I/PI	Microscope counting^6^
Detection limit^1^	>10^8^	ND^4^	10^7^	ND	5×10^5^	∼8×10^6^	10^5^	2.5×10^4^
Error^2^	ND	ND	30%∼40%	ND	20%∼30%	30%	<10%	10%∼20%
Time^3^	3∼5 h	2.5 h	1 h	20 min	50 min	2 h	20 min	∼48 h
Background signal	Low	Very strong	Near detection limit	Very strong	Low	Low	Low	No effect

1 Number of cells per 100 µl culture. Linear regression analysis (least squares regression method) was used to determine the detection limit of the assays. No linear-relationship (r <0.9) between cell numbers and corresponding measurement value was observed when the cell number is less than the detection limit. The values for linear regression analysis are the mean of three replicates.

2 Percentage of relative error from three time measurements.

3 Test time required for completing a 96-well plate assay.

4 ND: Not determined, due to weak signal of cells and/or strong background signal from BSK-H medium.

5 BSK-H medium was removed by washing prior to LIVE/DEAD BacLight assay.

6 Samples were stained with LIVE/DEAD BacLight assay.

### LIVE/DEAD BacLight assay

The commercial SYTO 9/PI (LIVE/DEAD BacLight) stain is commonly used for microscopic counting of live *B. burgdorferi* cells [3], [6], [13] and measuring the viability of environmental bacteria in water samples [14]. However, we found the SYTO 9/PI assay was not suitable for direct quantification of *B. burgdorferi* culture, due to the presence of large amounts of Bovine Serum Albumin (BSA) and phenol red in the BSK-H medium which strongly interfered with the BacLight assay (Figure 1A). When SYTO 9/PI was added to blank BSK-H medium or 50 mg/ml BSA solution (the same as the BSA concentration in BSK medium), the background green florescence intensity was even higher than that of the *B. burgdorferi* culture (Table 1). This problem suggests that the SYTO 9/PI assay could not quantify *B. burgdorferi* biomass directly in 96-well microtiter plate when cultured in the BSK-H medium. When the BSK-H medium was removed by washing prior to the actual analysis the viability assay worked better but a large number of *B. burgdorferi* cells were lost during the washing step, which severely compromises the utility of the assay for measuring the viability of *B. burgdorferi* when there is a large of number of samples to assay.

### Sytox Green/Hoechst 33342 assay

Recently, Cornell et al. developed a Sytox Green/Hoechst 33342 assay for assessing the viability of *B. burgdorferi* cells [15]. This assay was used to detect the dead *B. burgdorferi* by directly measuring fluorescence ratio using a microplate reader. We evaluated the Sytox Green/Hoechst 33342 assay in terms of sensitivity of detection and ability to measure live and dead cells. We found that the Sytox Green/Hoechst 33342 assay performed poorly in the 96-well plate format using known percentages of live and dead bacteria for ability to distinguish live and dead *B. burgdorferi* (Table 1). In addition, the linear ranges for detection were found to be between 8×10^6^ (detection limit) and 10^8^ spirochetes/ml for *B. burgdorferi* (Table 2). This linear range is higher than the spirochetes number of antibiotic-treated *B. burgdorferi* culture (<5×10^6^ spirochetes/ml in stationary phase culture) and thus may not be sensitive enough to measure the effect of antibiotic treatment. Another drawback of this assay was weak signal of the blue fluorescence of Hoechst 33342 dye, with the signal/noise ratio of Hoechst 33342 stain being 3.8, which is considerably lower than that of Sytox Green (17.7), PI (11.0) and SYBR Green I (25.0). The fluorescence was too weak to be recorded by the Spot slider color camera (Figure 1F). We could only capture the image of signal channel using the ORCA-R^2^ high resolution camera (Figure 1E). This weakness could not be overcome by increasing the concentration of Hoechst 33342 dye (data not shown), since the unbound Hoechst 33342 would emit green fluorescence at 510–540 nm which could produce false signal for Sytox Green (emission at the 523 nm) stain. The weak signal of live cells with this assay limited the minimum detectable level to 8×10^6^ spirochetes/ml with a significant error rate (30%) (Table 2).

### SYBR Green I/PI assay

SYBR Green I dye is commonly used for staining nucleic acids in gels in molecular biology techniques and is rarely used for bacterial viability assessment. Although SYBR Green I/PI assay has been used for viability assessment for other bacteria [16], [17], it has not been evaluated on *B. burgdorferi* as a viability stain. The mechanism of SYBR Green I/PI assay is similar to the SYTO 9/PI (BacLight) assay where SYBR Green I as a green permeant dye stains all cells whereas propidium iodide (PI) as an orange-red impermeant dye stains only dead or damaged cells with a compromised cell membrane. Thus live or viable cells with intact membrane will be stained only by SYBR Green I as green cells, while damaged or dead cells with a compromised membrane will be stained orange-red by PI. After trying various dyes including ethidium bromide (EB), PI, SYBR Green I, DAPI, and their combinations, we found that the SYBR Green I/PI combination was the best reagent for rapid viability assessment of *B. burgdorferi* (Table 1). It is interesting to note that in contrast to LIVE/DEAD BacLight viability assay which produced strong background (Figure 1A), the SYBR Green I/PI stain did not produce obvious background fluorescence with BSK-H medium (Figure 1B). In addition, we also optimized the SYBR Green I/PI viability assay with a range of concentrations (from 0.1×to 100 ×) of SYBR Green I dye, each in combination with varying concentrations of PI (from 0.1 mM to 5 mM) (data not shown). We found that the best ratio was 10 × SYBR Green I dye with 2 mM PI for viability assessment of *B. burgdorferi*.

**Figure 2:**
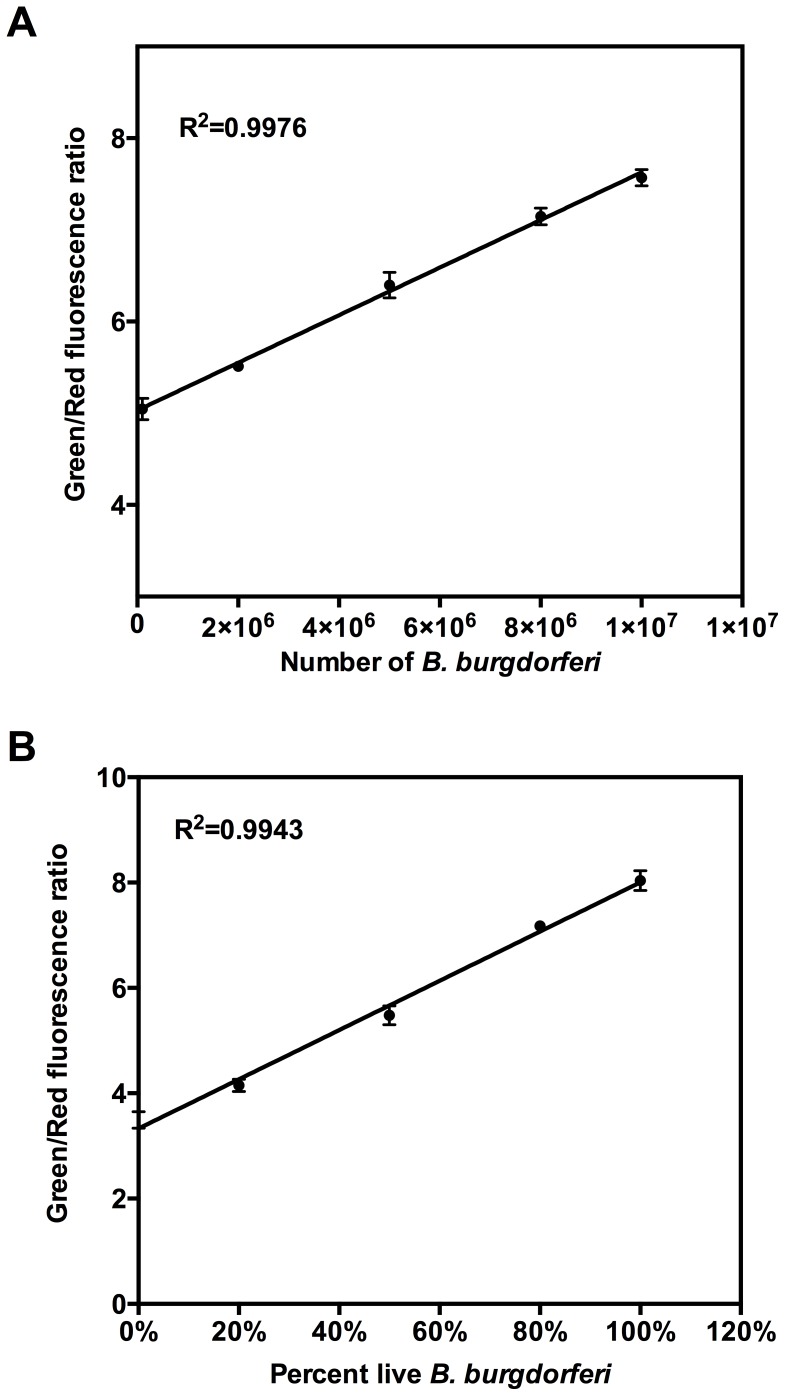
Linear relationship between the *B. burgdorferi* viability and Green/Red fluorescence ratio of the SYBR Green I/PI assay. (A) The linear relationship between the number of spirochetes and Green/Red fluorescence ratio. (B) Emission spectra of suspensions of various proportions of live and isopropyl alcohol-killed *B. burgdorferi* were obtained, and the Green/Red fluorescence ratios were calculated for each proportion of live/dead cells. The line is a least-square fit of the relationship between percentage of live bacteria and Green/Red fluorescence ratio.

We applied the SYBR Green I/PI assay to test serial dilutions of *B. burgdorferi* culture. It showed a linear relationship between the fluorescence ratio and the number of *B. burgdorferi* spirochetes when the concentration of spirochetes is higher than the 1×10^5^ spirochetes/ml (Figure 2A). The green and red fluorescence ratio of 1×10^5^ spirochetes/ml (5.04) was near the ratio of BSK medium (4.82), therefore we determined the quantitation limit of SYBR Green I/PI assay to be about 1×10^5^ spirochetes/ml in 96 well plate. To further validate the SYBR Green I/PI viability assay for *B. burgdorferi,* we used a mixture of live and dead *B. burgdorferi* cells in known proportions as standards. We initially attempted to prepare the known proportions of live and dead cells using heat-killed *B. burgdorferi* cells, but noted the heat-killed *B. burgdorferi* suspensions gelled or precipitated due to large amounts of serum or albumin in the BSK medium and could not be used as a source for known dead organisms for viability assessment. Then, we found that 70% isopropyl alcohol killed dead cells worked well and thus used such cells with live cells mixed in five different proportions (10^8^ bacteria/ml) in wells of the 96-well plate for the SYBR Green I/PI assay. The ratios of the integrated intensity of the portion of each spectrum at 535 nm (green) and 635 nm (red) for each bacterial suspension were calculated. The results are consistent with the results of fluorescence microscope counting, showing that the percentages of live bacteria correlated well with the ratio of green fluorescence to red fluorescence in a linear relationship for the SYBR Green/PI assay (Table 1, Figure 2B).

**Figure 3:**
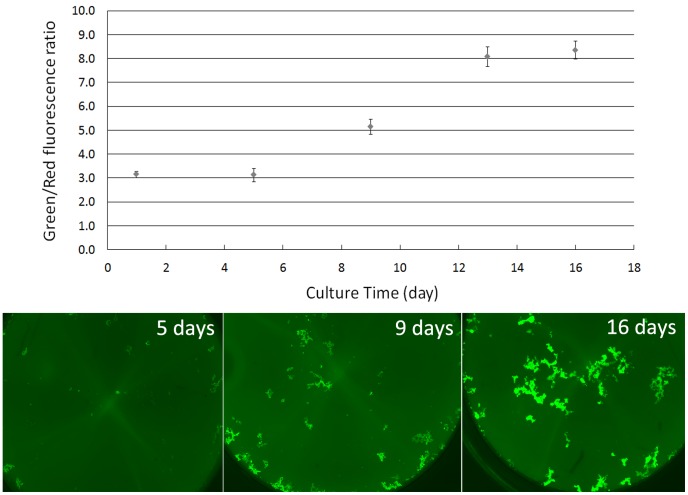
The Green/Red fluorescence ratios of *B. burgdorferi* biofilm measured by the SYBR Green I/PI assay at different culture times. The top panel shows the increase in Green/Red fluorescence ratios for biofilm growth over time, whereas the lower panel shows the corresponding microscopic images at different time points. All assays were run in triplicate.

Although it is not known whether the *B. burgdorferi* biofilm exists in**vivo, a recent study has shown that *B. burgdorferi* could form biofilm in vitro [4]. We therefore assessed the utility of the SYBR Green I/PI assay to measure the viability of the *B. burgdorferi* biofilm (Figure 1D, Figure 3). The fluorescence signals obtained in the biofilm assay showed good reproducibility, just as the signals obtained in the planktonic cultures (Figure 3). Using the SYBR Green I/PI assay, we could clearly plot the progression of *B. burgdorferi* biofilm formation overtime (Figure 3). It is worth noting that the biofim growth (Figure 3) is distinct from regular planktonic growth which could not form morphological structure on the well bottom and could not be observed under 20 × magnification used to view biofilm structures. Furthermore, the aggregated biofilm structure on the well bottom was hard to be dispersed in contrast to planktonic growth.

### Comparison of the performance of the SYBR Green/PI assay with other viability assays

Table 2 summarizes the comparison of the different viability assays. As can be seen, the MTT and XTT assays and the FDA assay had poor sensitivity or high background and could not be used as an efficient rapid method for viability assessment of *B. burgdorferi.* The commercial LIVE/DEAD BacLight kit worked poorly with very high background. The Sytox Green/Hoechst 33342 assay had lower sensitivity, higher error rate and was more time-consuming (2 hours) than the SYBR Green/PI assay. It is also difficult for microscope counting in that the weak blue fluorescence of live cells were hard to observe with triple filter (Figure 1F), which makes it inappropriate for high throughput screens. In contrast, the SYBR Green I/PI assay was overall the best assay for rapid viability measurement for *B. borgdorferi* compared with other assays in terms of reliability, sensitivity, low background, and speed. The SYBR Green I/PI assay could be performed directly without washing in just 20 min, with the lowest error rate of less than 10%, and a sensitivity of detection of 10^5^ bacteria, which is comparable to microscopic counting (Table 2). The microscopic counting with LIVE/DEAD BacLight stain had a comparable sensitivity as SYBR Green I/PI direct plate assay, but microscopy would be too tedious and time-consuming to be used for high throughput rapid viability assessment of *B. burgdorferi* in drug screens.

### Validation of the SYBR Green I/PI assay in antibiotic susceptibility testing

To assess if the SYBR Green I/PI assay could be used for antibiotic susceptibility testing, the *B. burgdorferi* was subjected to standard microdilution MIC determination with different concentrations of commonly used Lyme disease antibiotics doxycycline, amoxicillin, metronidazole and vancomycin in 96-well microtiter plate by plate reader HTS 7000 to determine the green to red fluorescence ratio (see Methods). As can be seen from Table 3, the MIC values determined by the SYBR Green I/PI assay were concordant with the MIC values reported in the literature [19].

**Table 3:** SYBR Green I/PI assay applied to MIC test on *Borrelia burgdorferi* ^a^.

Green/Red ratio	Drug concentrations used in MIC test (µg/ml)										MIC reported from literature (µg/ml)
	0.0	0.2	0.4	0.8	1.6	3.1	6.3	12.5	25.0	50.0	
Doxycycline	8.7 (+)^b^	**6.1 (−)^c^**	5.9 (−)	5.9 (−)	6.0 (−)	5.9 (−)	6.0 (−)	5.8 (−)	5.8 (−)	5.7 (−)	0.06 to 2^d^
Amoxicillin	9.0 (+)	**6.8 (−)^c^**	6.6 (−)	6.3 (−)	6.3 (−)	6.3 (−)	6.2 (−)	6.0 (−)	6.0 (−)	5.8 (−)	0.03 to 2^d^
Metronidazole	8.4 (+)	9.0 (+)	8.2 (+)	7.9 (+)	7.9 (+)	8.2 (+)	8.2 (+)	8.1 (+)	**6.1 (−)^c^**	6.0 (−)	0.06 to 32^e^
Vancomycin	8.0 (+)	7.4 (+)	**6.1 (−)^c^**	5.7 (−)	6.1 (−)	5.9 (−)	6.2 (−)	5.9 (−)	6.1 (−)	6.1 (−)	0.25 to 2^d^

a. The values in the table represent the mean Green/Red fluorescence ratios of three replicates. The results of triplicate microscope testing were correspondent.

b. Direct microscope counting results (+: grow, −: no grow) are shown in brackets.

c. MIC breakpoints determined by SYBR Green I/PI assay are shown in boldface.

d. Hunfeld, K. P. and V. Brade (2006). See reference [19].

e. Sapi, E., et al. See reference [6].

In addition, we assessed the ability of the SYBR Green I/PI assay to determine the viability of the *B. burgdorferi* stationary phase culture after antibiotic exposure to 1 mM each of doxycycline, amoxicillin, and metronidazole, in comparison with LIVE/DEAD BacLight assay and microscopic counting (used as a gold standard). We found that the SYBR Green I/PI assay showed good concordance with microscope counting for measuring viability of *B. burgdorferi* after antibiotic exposure. In contrast, the LIVE/DEAD BacLight assay did not show correlation with the microscopic counting (Table 4). These findings suggest the SYBR Green I/PI assay can be used for both MIC testing for growing *B. burgdorferi* and viability assessment for non-replicating bacteria after antibiotic exposure. Indeed, using this validated SYBR Green I/PI assay, we recently screened an FDA-approved drug library and successfully identified top 27 drug candidates from the 165 hits, including daptomycin, clofazimine, carbomycin, sulfamethoxazole, and cefoperazone that had excellent activity for *B. burgdoorferi* persisters [7].

**Table 4:** Validation of SYBR Green I/PI assay in comparison to microscopic counting for determining antibiotic susceptibility of *B. burgdorferi*.^1.^

Antibiotics	LIVE/DEAD (Ratio _Green/Red_)	SYBR Green I/PI (Ratio _Green/Red_)	Microscope counting (live %)^2^
Doxycycline	9.83	4.35	35%
Amoxicillin	10.18	5.76	66%
Metronidazole	10.34	7.12	81%
Drug-free control	10.32	8.85	95%

1 A 5-day *B. burgdorferi* culture was treated with 1 mM each of doxycycline, amoxicillin or metronidazole for 5 days, followed by staining with LIVE/DEAD assay and SYBR Green I/PI assay, or microscope counting (as a gold standard) to assess their capability for antibiotic susceptibility testing. The green/red ratios were obtained for LIVE/DEAD and SYBR Green/PI assays respectively in fluorescence measurement (see Methods). Each value is the mean of three replicates.

2 The percentage of live (green) spirochetes.

In summary, we have evaluated several bacterial viability assays and found the optimized SYBR Green I/PI assay to be the best method for viability assessment of *B. burgdorferi*. This study is the first to demonstrate the superiority of SYBR Green I/PI assay for *B. burgdorferi* viability assessment to all other current methods including the commercially available LIVE/DEAD BacLight assay in terms of accuracy, convenience, low background, and speed. The SYBR Green I/PI assay can be used as a convenient and sensitive test for rapid viability assessment in automated format for *B. burgdorferi* antibiotic susceptibility testing and for high-throughput drug screens.

## References

[1] CDC (2014) Lyme Disease. Available: http://www.cdc.gov/lyme/.

[2] Bacon RM, Kugeler KJ, Mead PS (2008) Surveillance for Lyme disease-United States, 1992–2006. MMWR 57: 1–9. 18830214

[3] Brorson O, Brorson SH, Scythes J, MacAllister J, Wier A, et al (2009) Destruction of spirochete Borrelia burgdorferi round-body propagules (RBs) by the antibiotic tigecycline. Proc Natl Acad Sci U S A 106: 18656–18661. 19843691 10.1073/pnas.0908236106 PMC2774030

[4] Sapi E, Bastian SL, Mpoy CM, Scott S, Rattelle A, et al (2012) Characterization of biofilm formation by Borrelia burgdorferi in vitro. PLoS One 7: e48277. 23110225 10.1371/journal.pone.0048277 PMC3480481

[5] Diterich I, Rauter C, Kirschning CJ, Hartung T (2003) Borrelia burgdorferi-induced tolerance as a model of persistence via immunosuppression. Infect Immun 71: 3979–3987. 12819085 10.1128/IAI.71.7.3979-3987.2003 PMC162029

[6] Sapi E, Kaur N, Anyanwu S, Luecke DF, Datar A, et al (2011) Evaluation of in-vitro antibiotic susceptibility of different morphological forms of Borrelia burgdorferi. Infect Drug Resistance 4: 97–113. 10.2147/IDR.S19201 PMC3132871 21753890

[7] Feng J, Wang T, Shi W, Zhang S, Sullivan D, et al.. (2014) Identification of Novel Activity against Borrelia burgdorferi Persisters Using an FDA Approved Drug Library. Emerg Microb Infect July 2, 2014: 3, e49; doi:10.1038/emi.2014.1053. 10.1038/emi.2014.53 PMC4126181 26038747

[8] Taylor JP, Wilson B, Mills MS, Burns RG (2002) Comparison of microbial numbers and enzymatic activities in surface soils and subsoils using various techniques. Soil Biol Biochem 34: 387–401.

[9] Sun Z, Zhang Y (1999) Spent culture supernatant of Mycobacterium tuberculosis H37Ra improves viability of aged cultures of this strain and allows small inocula to initiate growth. J Bacteriol 181: 7626–7628. 10601224 10.1128/jb.181.24.7626-7628.1999 PMC94224

[10] Gabrielson J, Hart M, Jarelov A, Kuhn I, McKenzie D, et al (2002) Evaluation of redox indicators and the use of digital scanners and spectrophotometer for quantification of microbial growth in microplates. J Microbiol Methods 50: 63–73. 11943359 10.1016/s0167-7012(02)00011-8

[11] Wang H, Cheng H, Wang F, Wei D, Wang X (2010) An improved 3-(4,5-dimethylthiazol-2-yl)-2,5-diphenyl tetrazolium bromide (MTT) reduction assay for evaluating the viability of Escherichia coli cells. J Microbiol Methods 82: 330–333. 20619304 10.1016/j.mimet.2010.06.014

[12] Mshana RN, Tadesse G, Abate G, Miorner H (1998) Use of 3-(4,5-dimethylthiazol-2-yl)-2,5-diphenyl tetrazolium bromide for rapid detection of rifampin-resistant Mycobacterium tuberculosis. J Clin Microbiol 36: 1214–1219. 9574679 10.1128/jcm.36.5.1214-1219.1998 PMC104802

[13] Peeters E, Nelis HJ, Coenye T (2008) Comparison of multiple methods for quantification of microbial biofilms grown in microtiter plates. J Microbiol Methods 72: 157–165. 18155789 10.1016/j.mimet.2007.11.010

[14] Boulos L, Prevost M, Barbeau B, Coallier J, Desjardins R (1999) LIVE/DEAD BacLight : application of a new rapid staining method for direct enumeration of viable and total bacteria in drinking water. J Microbiol Methods 37: 77–86. 10395466 10.1016/s0167-7012(99)00048-2

[15] Cornell KA, Primus S, Martinez JA, Parveen N (2009) Assessment of methylthioadenosine/S-adenosylhomocysteine nucleosidases of Borrelia burgdorferi as targets for novel antimicrobials using a novel high-throughput method. J Antimicrob Chemother 63: 1163–1172. 19376840 10.1093/jac/dkp129 PMC2734086

[16] Barbesti S, Citterio S, Labra M, Baroni MD, Neri MG, et al (2000) Two and three-color fluorescence flow cytometric analysis of immunoidentified viable bacteria. Cytometry 40: 214–218. 10878564

[17] Cerca F, Trigo G, Correia A, Cerca N, Azeredo J, et al (2011) SYBR green as a fluorescent probe to evaluate the biofilm physiological state of Staphylococcus epidermidis, using flow cytometry. Can J Microbiol 57: 850–856. 21950962 10.1139/w11-078

[18] Zambrano MC, Beklemisheva AA, Bryksin AV, Newman SA, Cabello FC (2004) Borrelia burgdorferi binds to, invades, and colonizes native type I collagen lattices. Infect Immun 72: 3138–3146. 15155615 10.1128/IAI.72.6.3138-3146.2004 PMC415685

[19] Hunfeld KP, Brade V (2006) Antimicrobial susceptibility of Borrelia burgdorferi sensu lato: what we know, what we don't know, and what we need to know. Wien Klin Wochenschr 118: 659–668. 17160604 10.1007/s00508-006-0693-z

